# Surgically treated solitary giant gluteal and retroperitoneal neurofibroma: a case report

**DOI:** 10.1186/s12957-016-0880-y

**Published:** 2016-04-27

**Authors:** Xiang-qian Shen, Hui Shen, Shou-cheng Wu, Ying Lv, Hui Lu, Xiang-jin Lin

**Affiliations:** Department of Hand Surgery and Microsurgery Center, The First Affiliated Hospital, College of Medicine, ZheJiang University, HangZhou, China; The Children’s Hospital, Zhejiang University School of Medicine, HangZhou, China; Department of Orthopaedics, The First Affiliated Hospital, College of Medicine, ZheJiang University, 79# Qingchun Road, HangZhou, 310003 ZheJiang province People’s Republic of China

**Keywords:** Neurofibromatosis type 1, Giant neurofibroma, Surgical treatment, Gluteal, Retroperitoneal

## Abstract

**Background:**

Giant neurofibromas in patients with neurofibromatosis type 1 involve multiple regions and are often difficult to surgically extirpate. However, surgical intervention is the most effective means for improving quality of life. The case reported herein is unique in that it involves a giant neurofibroma, involving the patient’s peritoneal and pelvic cavities, retroperitoneal space, and buttock, which was causing compressive displacement of abdominal and pelvic organs. A challenging surgical intervention was required to accomplish near-total resection to relieve organ compression while preserving visceral and genitoanal function.

**Case presentation:**

The case reported is of a patient presenting with a solitary giant retroperitoneal neurofibroma that threatened to obliterate both peritoneal and pelvic cavities and protruded conspicuously into the right gluteal region. The enormous dumbbell-shaped mass was surgically removed in three parts. Postoperative pathology studies confirmed a diagnosis of neurofibroma. Follow-up computed tomography images taken three months postoperatively revealed residual tumor in the perianal region. The patient’s quality of life had measurably improved on follow-up at eight months.

**Conclusions:**

Surgical intervention in such extraordinary circumstances of a giant neurofibroma causing compressive displacement of critical organs reduces tumor burden, restores appearance and function of patient’s body and internal organs, and improves the patient’s quality of life.

## Background

Neurofibromatosis type 1 (NF1, von Recklinghausen’s disease) is an autosomal-dominant inheritable syndrome attributable to genetic mutations in the coding of neurofibromin [1]. Although generally benign, the expansile neurofibromas characteristic of NF1 readily displace contiguous organs, resulting in highly visible malformations and other dysfunctions [2]. Intrapelvic neurofibromas are often diagnosed at an advanced stage and therefore are difficult to surgically extirpate. Described herein is a patient presenting with a solitary giant retroperitoneal neurofibroma that threatened to obliterate both peritoneal and pelvic cavities and protruded conspicuously into the right gluteal region.

## Case presentation

### Case report

**Fig. 1:**
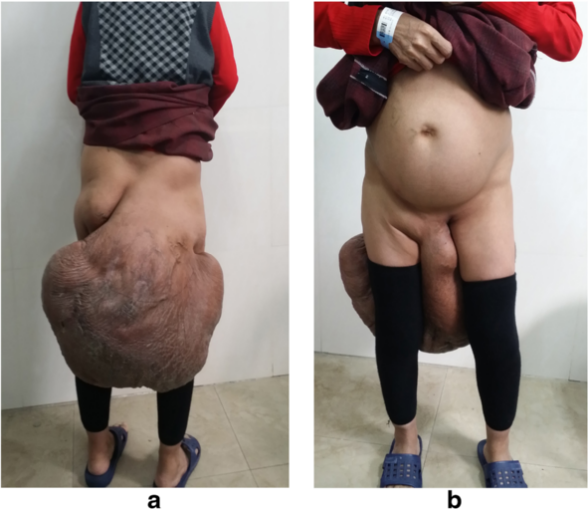
**a**, **b** A 45-year-old female patient was hospitalized for a sizeable tumor of the right buttock

This 45-year-old female patient was hospitalized for a sizeable tumor of the right buttock (Fig. 1) that impaired daily life and social activities. A diagnosis of neurofibroma was established by incisional biopsy.

**Fig. 2:**
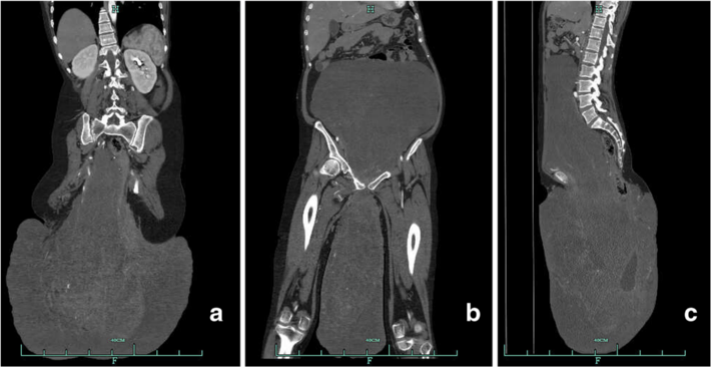
**a**–**c** CT revealed an enormous dumbbell-shaped mass (68.3 cm superoinferiorly) occupying the mid-abdomen

Contrast-enhanced computed tomography (CT) revealed an enormous dumbbell-shaped mass (68.3 cm superoinferiorly), occupying the mid-abdomen, pelvic cavity, and buttock. The rectum, uterus, and urinary bladder were compressed and shifted to the left (Fig. 2). On CT angiography, the tumor was supplied in part by the right internal iliac artery and by the right femoral and left internal iliac arteries.

#### Operative and postoperative course

The patient was placed in lithotomy position for the two-part tumor resection. First, a 20-cm midline incision was made from the umbilicus to pubic region, exposing a bulky encapsulated tumor of the abdomen and pelvis. The mass displayed solid and cystic areas, reaching a diameter of 30 cm. It was soft to the touch and grossly intact, completely displacing the peritoneal and pelvic contents. The bowel, uterus, and bladder were compressed and shifted laterally. The ureters and bladder were secured while freeing the encapsulated edge of the mass and severing its pelvic floor attachment, thus enabling *en bloc* abdominopelvic tumor removal. The protuberant skin-covered gluteal component, still communicating with the pelvic tumor, was incised along its base to maximize excision and skin procurement.

**Fig. 3:**
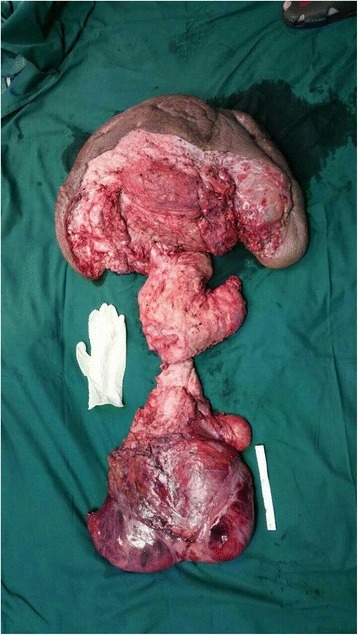
The excised mass measured 50 cm across and weighed ~25 kg

**Fig. 4:**
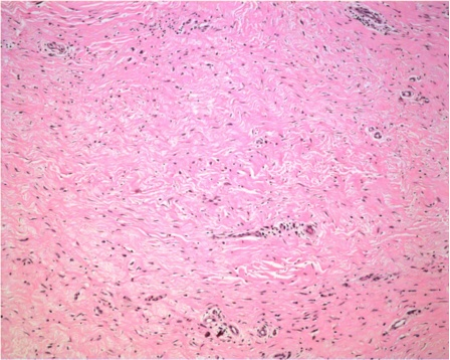
Postoperative pathology studies confirmed a diagnosis of neurofibroma. (HE staining, ×200)

Having no clear boundary, the narrowest segment of the dumbbell-shaped mass also involved the vagina and anus, creating compressive displacement (to the left) and preventing complete excision without mutilation. The remainder of this segment was isolated along its border and removed. Ultimately, the excised mass measured 50 cm across and weighed ~25 kg (Fig. 3). Postoperative pathology studies confirmed a diagnosis of neurofibroma (positive: vimentin, CD34; negative: S-100, smooth muscle actin [SMA], desmin, CD117, cytokeratin [CK], epithelial membrane antigen [EMA]) (Fig. 4).

**Fig. 5:**
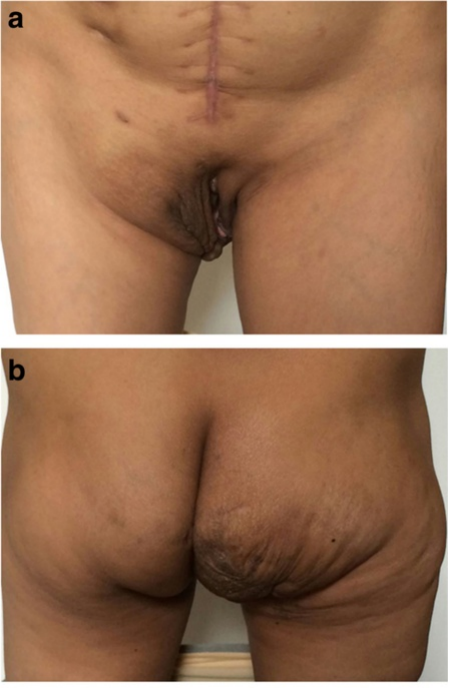
**a**, **b** Gluteal and perineal regions were satisfactory, and the patient’s quality of life had measurably improved

Postoperatively, the patient became febrile. B-scan ultrasonography indicated a seroperitoneum of moderate degree that was evacuated on postoperative day 6 via B-scan ultrasound-guided paracentesis. The fever thereafter abated. Three months postoperatively, a residual perianal tumor was presumptively identified in follow-up CT images. The gluteal and perineal regions were otherwise satisfactory, and the patient’s quality of life had measurably improved (Fig. 5).

### Discussion

Infiltrative neurofibromas (with ill-defined borders) cannot be treated radically [3]. The neurofibromas can reoccur after surgical intervention [4]. However, surgical intervention is the most effective means for improving quality of life [5]. A giant tumor of this nature, involving the peritoneal and pelvic cavities, retroperitoneal space, and buttock, is truly a surgical challenge. Although resection is the accepted practice, a number of surgeons find this controversial and prefer clinical observation [1]. As a less aggressive measure, the gluteal mass alone could have been targeted, but our multidisciplinary team felt that near-total resection was needed to relieve abdominal organ compression. Because radical resection was prohibitive, our objective was visceral and genitoanal functional preservation. The resultant gluteal and perineal defects were too extensive for reparative local flaps; therefore, skin resected with the tumor was reserved for reconstruction purposes.

## Conclusions

This report validates the use of surgical intervention to reduce tumor burden, restore appearance and function, and improve quality of life in such extraordinary circumstances.

### Consent

Written informed consent was obtained from the patient for publication of this case report and any accompanying images. A copy of the written consent is available for review by the Editor-in-Chief of this journal.

## Abbreviations

CK: cytokeratin
CT: computed tomography
EMA: epithelial membrane antigen
NF1: neurofibromatosis type 1
SMA: smooth muscle actin
